# Pan-cancer analysis of ADAMs: A promising biomarker for prognosis and response to chemotherapy and immunotherapy

**DOI:** 10.3389/fgene.2023.1105900

**Published:** 2023-04-04

**Authors:** Bo Ma, Riyue Yu

**Affiliations:** Department of Stomatology, Beijing Shijitan Hospital, Capital Medical University, Beijing, China

**Keywords:** ADAMs, pan-cancer, TME, prognosis, chemotherapy, immunotherapy

## Abstract

**Background:** Members of a disintegrin and metalloproteinase (ADAM) family play a vital role in cancer development. However, a comprehensive analysis of the landscape of the ADAM family in pan-cancer remains to be performed.

**Methods:** The correlation of the expression level and prognostic value with ADAMs in a pan-cancer cohort and the relationship between ADAMs and the stemness score, tumour microenvironment (TME), chemotherapy-related drug sensitivity, immune subtype, and immunotherapy outcome were investigated.

**Results:** ADAMs were differentially expressed between tumour and para-carcinoma tissues in the pan-cancer cohort, and the expression of ADAMs was significantly correlated with patient prognosis. Furthermore, ADAMs were significantly correlated with the stromal score and immune score based on the TME analysis. Additionally, ADAMs were also correlated with DNAss and RNAss in the pan-cancer cohort. On investigating the CellMiner database, ADAMs were revealed to be significantly correlated with the sensitivity of various drugs, including raloxifene and tamoxifen. Moreover, in the IMvigor210 and GSE78220 cohorts, ADAMs were correlated with immunotherapy response and immune activation genes. Furthermore, quantitative real-time polymerase chain reaction (qRT-PCR) and immunohistochemistry (IHC) were utilised to determine the differential level of ADAM9 in cancer and para-carcinoma tissues in patients’ samples.

**Conclusion:** This study elucidates the importance of ADAMs in cancer progression and lays a foundation for further exploration of ADAMs as potential pan-cancer targets.

## 1 Introduction

The extracellular matrix (ECM) is a macromolecular structure consisting of the basement membrane (BM) and intercellular substance (Padhi and Nain, 2020). The ECM constitutes a complex network structure that supports and connects tissue structures and regulates tissue formation and cellular physiological activities (Valdoz et al., 2021).

The ECM acts as an important bio-barrier for tumour local or distant metastasis. Tumour cells secrete proteolytic enzymes to degrade the ECM, thereby creating a local lysis zone for cancer cell intravasation and extravasation (Najafi et al., 2019). Generally, cancer cells with a high degree of malignancy have a strong proteolytic effect, which can erode and destroy the membrane and promote metastasis. The enzymes involved in this process are mainly serine proteases, such as plasminogen activator (PA) and metalloproteinase (MP), which include collagenase IV, matrix-degrading enzymes, and hyaluronidase (Mohan et al., 2020). Various secreted proteins, including cross-linkers, modifying enzymes, and proteases and their inhibitors, regulate ECM homoeostasis. Thus, the activation or inhibition of the aforementioned proteins promotes the proliferation and invasion of cancer cells, which suggests their potential as therapeutic targets in cancer treatment (Walker et al., 2018).

In addition to degradation, the turnover of the matrix is also significantly important for ECM homoeostasis (Huang et al., 2021). The substrate turnover of the matrix is regulated by various enzyme families. The “normal” stroma degradation followed by an increase in the stromal turnover of the tumour promotes the replacement by the tumour stroma, reinforces aggressive features, and removes physical barriers (e.g., the basement membrane), consequently favouring the malignancy and metastasis of tumours (Nissen et al., 2019). As an important ligand pool of growth factors, the degradation and turnover of the ECM could activate the binding growth factors and induce intracellular signalling responses. Moreover, the ECM not only includes enzymes such as growth factors, chemokines, and cytokines but also is a repository for inorganic molecules (Girigoswami et al., 2021). During matrix remodelling, divalent cations like calcium ions are activated to facilitate calcium transport, which further induces the activation of matrix metalloproteinases (MMPs), a disintegrin and metalloproteinases (ADAMs), and ADAMs with thrombospondin motifs (ADAMTSs), which constitute the calcium-dependent, zinc-containing thyroxine superfamily endopeptidases (Theret et al., 2021).

The ADAM family can be divided into trans-model ADAM and secretory ADAM components based on structural differences (Saha et al., 2019). The genomic distribution of ADAMs is presented in Table 1. The pre-control region ensures that the metalloprotease domain is inactive, while the catalytic site is activated by the activation of the cysteine switch mechanism. The furin recognition site (RXXR sequence) is located between the pre-control region and the metalloprotease domain, which is involved in the intracellular activation of many ADAM family members in a trans-Golgi network by a furin-like preprotein convertase. The active site of the metalloprotease domain of ADAM molecule with proteolytic activity contains a common “HEXGH” conserved sequence, whose alteration will result in the loss of proteolytic activity (Malemud, 2019). Although the ADAM family has been extensively studied, little is known about the specificity of ADAM substrates, which are hypothesised to be determined by disintegrin and cysteine domains, especially the interaction of a substrate and enzyme (Heib et al., 2020). The main function of ADAM molecules is to mediate “extracellular domain shedding,” a post-translational mechanism. ADAMs can induce proteolytic processing to release the membrane-attached proteins and activate the cleaved molecules, which are involved in growth factor signalling, cell migration, cell adhesion, and other aspects (Camodeca et al., 2019). Studies report that ADAM is abnormally upregulated and downregulated in various malignant tumour tissues, such as lung cancer, liver cancer, and colon cancer (Li et al., 2018; Jin et al., 2020; Du et al., 2022). Such abnormal expression promotes the proliferation of tumour cells and participation in tumour angiogenesis by regulating intercellular adhesion and degrading the intercellular substance.

**TABLE 1:** Chromosome location of ADAMs.

ADAM	Chromosome location
ADAM2	8p11.22
ADAM7	8p21.2
ADAM8	10q26.3
ADAM9	8p11.22
ADAM10	15q21.3
ADAM11	17q21.31
ADAM12	10q26.2
ADAM15	1q21.3
ADAM17	2p25.1
ADAM18	8p11.22
ADAM19	5q33.3
ADAM20	14q24.2
ADAM21	14q24.2
ADAM22	7q21.12
ADAM23	2q33.3
ADAM28	8p21.2
ADAM29	4q34.1
ADAM30	1p12
ADAM32	8p11.22
ADAM33	20p13

In this study, we aimed to investigate the potential effects and mechanisms of ADAM families for pan-cancer analysis across 33 distinct tumours by integrating bulk RNA-seq, tumour mutation burden (TMB), and clinicopathological parameters in The Cancer Genome Atlas (TCGA) datasets. Moreover, we further used ESTIMATE and CIBERSORT to explore the correlation of ADAMs with immune cell infiltration and TME status. Moreover, the sensitivity of distinct FDA-approved drugs to target cancers was also examined through the CellMiner database. Furthermore, as ADAMs were significantly correlated with TME infiltration, the sensitivity of immune checkpoint inhibitor (ICI)-based immunotherapy and the association between the expression of ADAMs and the outcomes of patients treated with ICIs were explored in IMvigor210 and GSE78220 cohorts, and we found that ADAM19 was strikingly correlated with ICI immunotherapy response. Moreover, we also found that ADAMs, especially ADAM9, were strikingly correlated with the sensitivity of FDA-approved drugs. Considering the correlation of all ADAMs with drug sensitivity and immunotherapy response, we finally verified that ADAMs were significantly associated with all types of cancers and might be the novel targets for chemotherapy and immunotherapy.

## 2 Materials and methods

### 2.1 Identification of ADAMs in TCGA pan-cancer cohort

RNA-seq (FPKM) gene expression data were downloaded from the open access database UCSC Xena (http://xena.ucsc.edu) and were transformed into transcripts per kilobase million (Wagner et al., 2012). Clinical information of the samples and pathological information, including immune subtypes and stemness scores (RNA-based, RNAss; DNA methylation, DNAss), for all these cancers were also acquired from UCSC Xena (Miao et al., 2020). For pan-cancer TCGA analysis, a total of 20 ADAM (Supplementary Table S1) expression levels were extracted (Supplementary Table S2), and the differences in para-carcinoma and tumour tissue samples were evaluated using Student’s *t*-test. Importantly, the number of normal samples of some cancer types was less than five; hence, these cancer types were excluded to prevent a statistical error. The box plots and heat maps were designed using “ggpubr” and “pheatmap” in R. The ADAM internal correlation was performed using “corrplot” in R.

### 2.2 Survival analyses of ADAMs in pan-cancers

Kaplan–Meier survival curves and log-rank test (*p*-value cut-off point 0.05) were used for the survival analysis. The cut-off was selected based on the average expression level of ADAMs in each tumour sample, and the patients were divided into high-expression and low-expression groups. Survival curves were plotted using the R packages “survminer” and “survival.” Furthermore, Cox analysis was performed to determine the relationship between ADAMs and cancer prognosis. Finally, the forest plot was drawn using the R packages “survival” and “forestplot.”

### 2.3 Correlation of ADAMs with the TME and stemness score in pan-cancers

The stromal scores and immune cell scores were calculated using “ESTIMATE” and “limma” in R for evaluating the stromal cell and immune cell infiltrating levels. Spearman’s correlations were used to analyse the correlation between ADAMs and RNAss/DNAss, and the R package “corrplot” was used to visualise the results.

### 2.4 Correlation of ADAMs with chemotherapy-related drug sensitivity and the immune subtype

We acquired the chemotherapy-related drug sensitivity data from an open access database CellMiner (https://discover.nci.nih.gov/cellminer/loadDownload.do). Moreover, “limma” and “ggplot2” were used for data analysis and visualisation. Th immune subtypes of the samples were acquired from UCSC Xena. Furthermore, the correlation between immune subtypes and ADAMs was analysed by “limma” and “reshape2” in R.

### 2.5 Correlation of ADAMs and immunotherapy

The immunotherapy data were obtained from the IMvigor210 and GSE78220 datasets. The treatment outcomes are shown in Supplementary Table S3. Visualisation and response analysis of the result were processed using “ggpubr,” “ggplot2,” and “limma” in R.

### 2.6 Correlation of ADAM19 with TMB, microsatellite instability, and immune activation-related genes

The TMB and microsatellite instability (MSI) were calculated using TCGA somatic mutation data. A radar legend was established to show the relationship between ADAM19 and the TMB and MSI using Spearman’s correlation analysis. Additionally, the co-expression of ADAM19 and immune activation-related genes was further analysed.

### 2.7 Tissue specimens and immunohistochemistry

We collected 18 paired KIRC and BLCA samples from Beijing Friendship Hospital, Capital Medical University (Beijing, China), between June 2022 and October 2022. The Institutional Research Ethics Committee approved the sample collection (No. 2021-P2-159). All samples were pathologically confirmed to be KIRC or BLCA. The antibody of ADAM9 was acquired from ABclonal (A22058, Wuhan, China).

### 2.8 Total RNA extraction, reverse transcription, and quantitative real-time polymerase chain reaction

The RNeasy Plus Mini Kits (74136, QIAGEN, Germany) were used to extract the total RNA of the samples. Subsequently, the quality of the extracted RNA was examined using NanoDrop (NP80, Implen, Germany). Following this, the ReverTra Ace qPCR RT Kit (FSQ-201, TOYOBO, Japan) was used for further cDNA synthesis. Finally, reverse and forward primers designed by us and iQ^TM^ SYBR^®^ Green Supermix (1708880, Bio-Rad, United States) were mixed to perform qRT-PCR. The expression of the targeted genes was normalised using the expression of GAPDH (Schmittgen and Livak, 2008).

### 2.9 Statistical analyses

Statistical significance between two groups was tested using Student’s *t*-test. For variables that fall into more than three groups, a one-way analysis of variance or the Kruskal–Wallis test was used, depending on the type of data. Kaplan–Meier (KM) curves were used to calculate and visualise the survival rates, and the log-rank test was used to test whether differences were significant. Correlation coefficients were calculated using Spearman’s correlation analysis. A univariate Cox proportional hazards model was used to determine the timing of the variables and whether they were independent predictors. Statistical significance was set at *p* < 0.05.

## 3 Results

### 3.1 The landscape of ADAMs in the pan-cancer cohort

**FIGURE 1:**
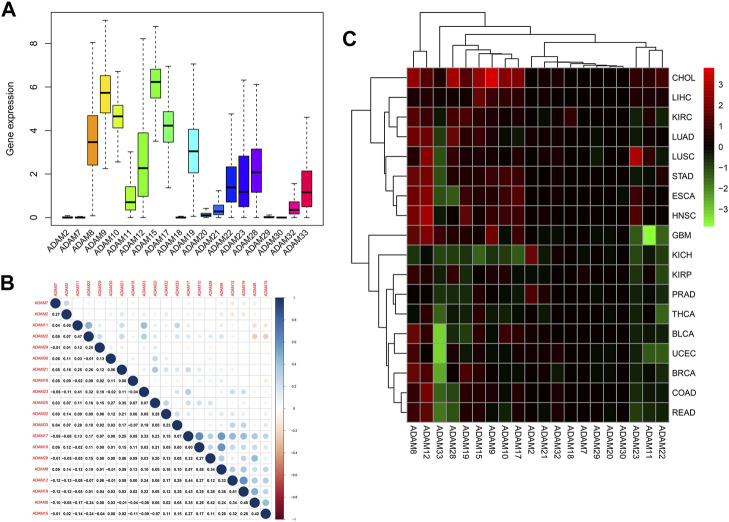
Expression levels and correlations of ADAM family genes in different types of cancer in TCGA. **(A)** Over-expression or under-expression of the ADAM family in various types of cancer. **(B)** Expression data from TCGA showing the ADAM family expressed in different types of cancer. The colour of each small rectangle represents high or low expression of ADAM family genes in each cancer. Red and green colours indicate high and low expression, respectively. **(C)** Correlations between ADAM family genes. Blue dots represent positive correlations, and red dots represent negative correlations. ACC, adrenocortical carcinoma; BLCA, bladder urothelial carcinoma; BRCA, breast invasive carcinoma; CESC, cervical squamous cell carcinoma and endocervical adenocarcinoma; CHOL, cholangiocarcinoma; COAD, colon adenocarcinoma; DLBC, diffuse large B-cell lymphoma; ESCA, oesophageal carcinoma; GBM, glioblastoma multiforme; HNSC, head and neck squamous cell carcinoma; KICH, kidney chromophobe; KIRC, kidney renal clear cell carcinoma; KIRP, kidney renal papillary cell carcinoma; LAML, acute myeloid leukaemia; LGG, brain lower-grade glioma; LIHC, liver hepatocellular carcinoma; LUAD, lung adenocarcinoma; LUSC, lung squamous cell carcinoma; MESO, mesothelioma; OV, ovarian serous cystadenocarcinoma; PAAD, pancreatic adenocarcinoma; PRAD, prostate adenocarcinoma; READ, rectum adenocarcinoma; SKCM, skin cutaneous melanoma; STAD, stomach adenocarcinoma; TGCA, testicular germ-cell tumour; THCA, thyroid carcinoma; THYM, thymoma; UCEC, uterine corpus endometrial carcinoma; UVM, uveal melanoma.

Figure 1A displays the expression of ADAMs in 33 types of cancers. Moreover, a series of genes in ADAMs, namely, ADAM8, ADAM9, ADAM10, ADAM11, ADAM12, ADAM17, ADAM19, ADAM21, ADAM22, ADAM23, ADAM28, ADAM32, and ADAM33, were highly expressed among all types of cancers. Additionally, the correlation between different ADAMs was also explored (Figure 1B), with ADAM8 and ADAM22 exhibiting the most significant positive correlation, whereas ADAM9 and ADAM10/ADAM17 exhibiting the most negative correlation. We further explored the expression of all ADAMs in 33 cancers (Figure 1C). ADAM9 was observed to be highly expressed in CHOL, whereas ADAM33 had a significantly lower expression in pan-cancers, especially in BLCA and UCEC (Figure 1C).

**FIGURE 2:**
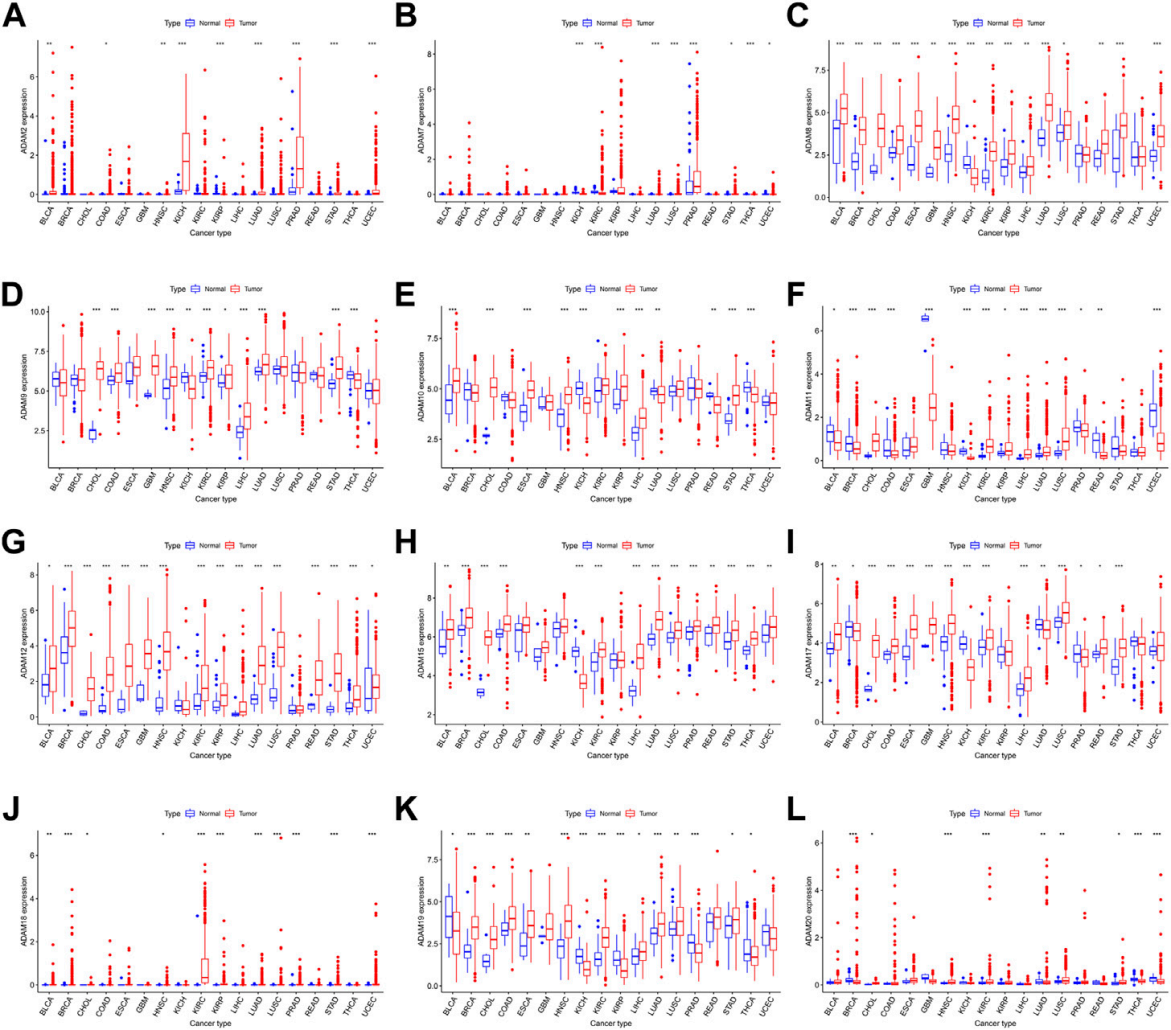
ADAM family expression levels in different cancer and para-carcinoma tissues. **(A)** Differential expression of ADAM2, **(B)** differential expression of ADAM7, **(C)** differential expression of ADAM8, **(D)** differential expression of ADAM9, **(E)** differential expression of ADAM10, **(F)** differential expression of ADAM11, **(G)** differential expression of ADAM12, **(H)** differential expression of ADAM15, **(I)** differential expression of ADAM17, **(J)** differential expression of ADAM18, **(K)** differential expression of ADAM19, and **(L)** differential expression of ADAM20. The red rectangle box represents gene expression levels in tumour tissue, and the blue rectangle box represents normal tissue. *p < 0.05, **p < 0.01, and ***p < 0.001. Cancer names in red indicate high expression, and cancer names in blue indicate low expression of the corresponding ADAM family gene.

We also extracted the expression of ADAMs in TCGA database using R software, and the resultant expression is presented in Supplementary Table S2. ADAMs were differentially expressed among all cancers and para-carcinoma tissues (Figure 2; Supplementary Figure S1).

### 3.2 Correlation of ADAMs and prognosis in the pan-cancer cohort

**FIGURE 3:**
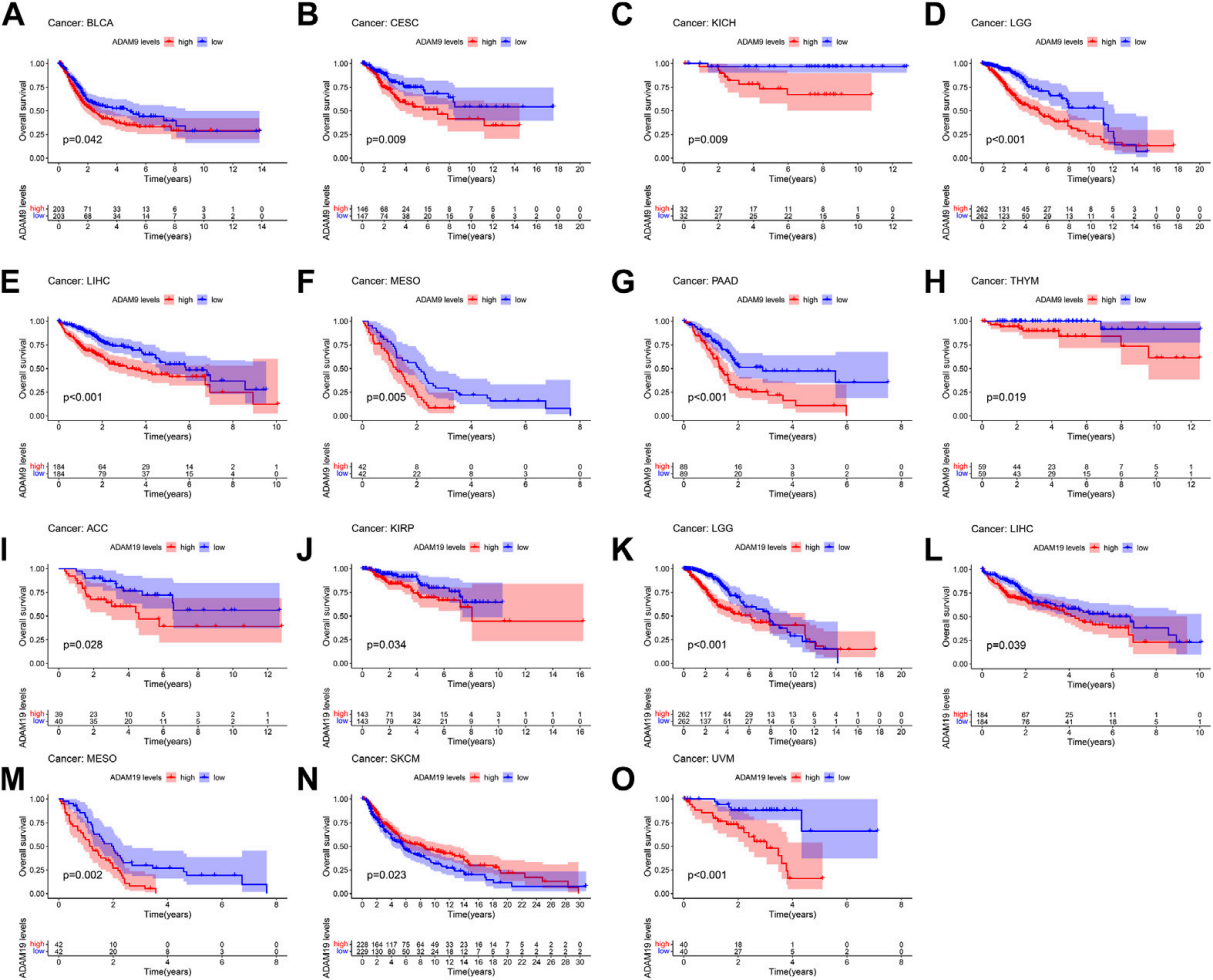
Kaplan–Meier survival curve comparison of high/low expression of ADAM family genes in pan-cancer. **(A)** Survival curves of ADAM9 in BLCA (N = 406), **(B)** survival curves of ADAM9 in CESC (N = 293), **(C)** survival curves of ADAM9 in KICH (N = 64), **(D)** survival curves of ADAM9 in LGG (N = 524), **(E)** survival curves of ADAM9 in LIHC (N = 368), **(F)** survival curves of ADAM9 in MESO (N = 84), **(G)** survival curves of ADAM9 in PAAD (N = 177), **(H)** survival curves of ADAM9 in THYM (N = 118), **(I)** survival curves of ADAM19 in ACC (N = 79), **(J)** survival curves of ADAM19 in KIRP (N = 286), **(K)** survival curves of ADAM19 in LGG (N = 524), **(L)** survival curves of ADAM19 in LIHC (N = 368), **(M)** survival curves of ADAM19 in MESO (N = 84), **(N)** survival curves of ADAM19 in SKCM (N = 457), and **(O)** survival curves of ADAM19 in UVM (N = 80).

**FIGURE 4:**
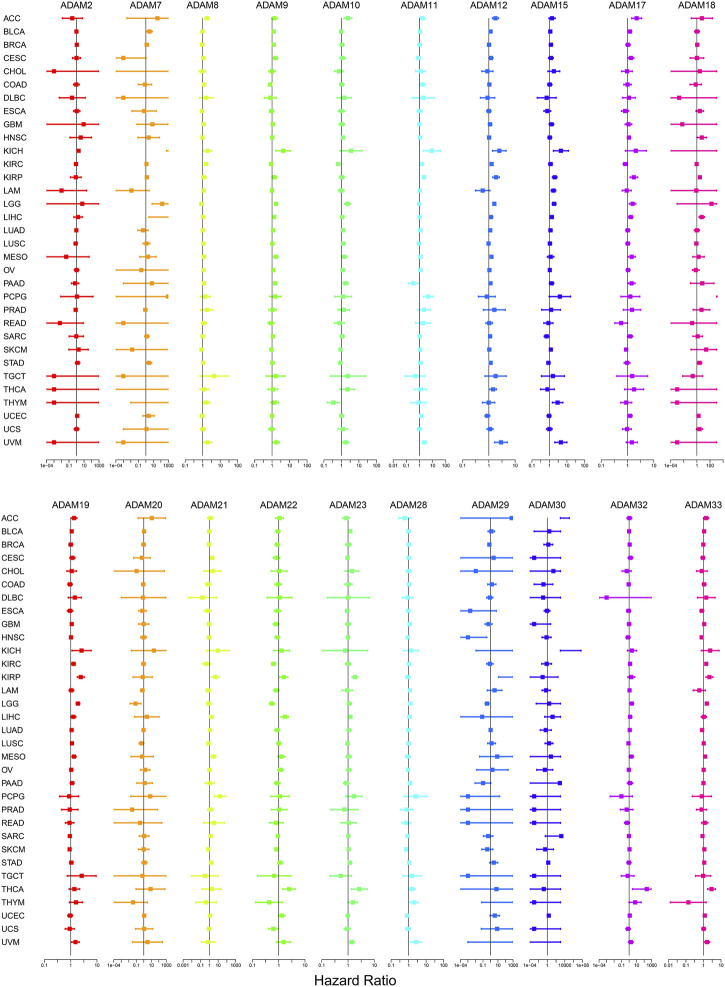
Cox regression analysis of the correlation between ADAM family expression and survival. Lines with different colours represent the risk values for different genes within the tumour, with a hazard ratio <1 indicating low risk and a hazard ratio >1 indicating high risk.

The number of normal samples of some cancer types was less than five in our study; hence, these cancers were excluded to prevent a statistical error, and a total of 28 cancers were contained in this study. We subsequently analysed the correlation between the expression of ADAMs and prognostic data. The Kaplan–Meier survival curves of ADAM9 and ADAM19 are shown in Figure 3, and the *p*-values of the survival analysis of other ADAMs are listed in Supplementary Table S4. Among the analysed ADAMs, ADAM9 had a negative effect on BLCA (N = 406, *p* = 0.042; Figure 3A), CESC (N = 293, *p* = 0.009; Figure 3B), KICH (N = 64, *p* = 0.009; Figure 3C), LGG (N = 524, *p* < 0.001; Figure 3D), LIHC (N = 368, *p* < 0.001; Figure 3E), MESO (N = 84, *p* = 0.005; Figure 3F), PAAD (N = 177, *p* < 0.001; Figure 3G), and THYM (N = 118, *p* = 0.019; Figure 3H). Furthermore, ADAM19 also had a negative effect on ACC (N = 79, *p* = 0.028; Figure 3I), KIRP (N = 286, *p* = 0.034; Figure 3J), LGG (N = 524, *p* < 0.001; Figure 3K), LIHC (N = 368, *p* = 0.039; Figure 3L), MESO (N = 84, *p* = 0.002; Figure 3M), and UVM (N = 80, *p* < 0.001; Figure 3O), while ADAM19 appeared to induce a protective effect on SKCM (N = 457, *p* = 0.023; Figure 3N). Furthermore, we investigated the prognostic risk of ADAMs in pan-cancers using Cox regression analysis, which revealed results consistent with that of the Kaplan–Meier survival curves (Figure 4).

### 3.3 Association of ADAMs with the TME and stemness score in pan-cancers

**FIGURE 5:**
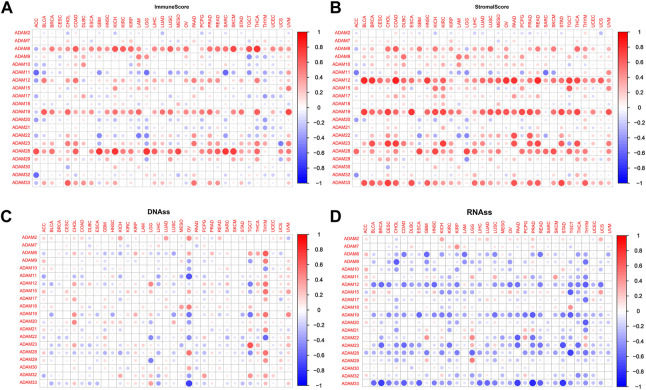
Correlation of ADAM family gene expression with the tumour microenvironment and stemness score in pan-cancer. **(A, B)** ADAM family gene expression correlates with various mesenchymal and immune cancer scores. Red dots indicate a positive correlation between tumour gene expression and mesenchymal score, and green dots indicate a negative correlation between tumour gene expression and mesenchymal score. **(C, D)** Correlation of ADAM family expression with RNAss and DNAss in pan-cancer. Red dots indicate a positive correlation between tumour gene expression and immune score, and blue dots indicate a negative correlation between tumour gene expression and immune score.

We explored the relationship between the expression of ADAMs and the TME in pan-cancers. The immune scores and stromal scores were significantly positively correlated with the expression of ADAMs (Figures 5A, B). Furthermore, significant negative or positive correlations were found between ADAMs and RNAss (Figure 5C). A correlation between ADAMs and DNAss (Figure 5D) was also found in pan-cancers (Supplementary Table S5).

**FIGURE 6:**
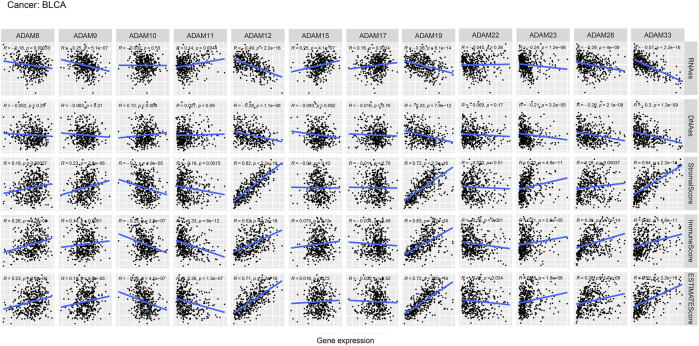
Correlation analysis of ADAM family gene expression with RNAss, DNAss, stromal score, immune score, and ESTIMATE score in BLCA.

**FIGURE 7:**
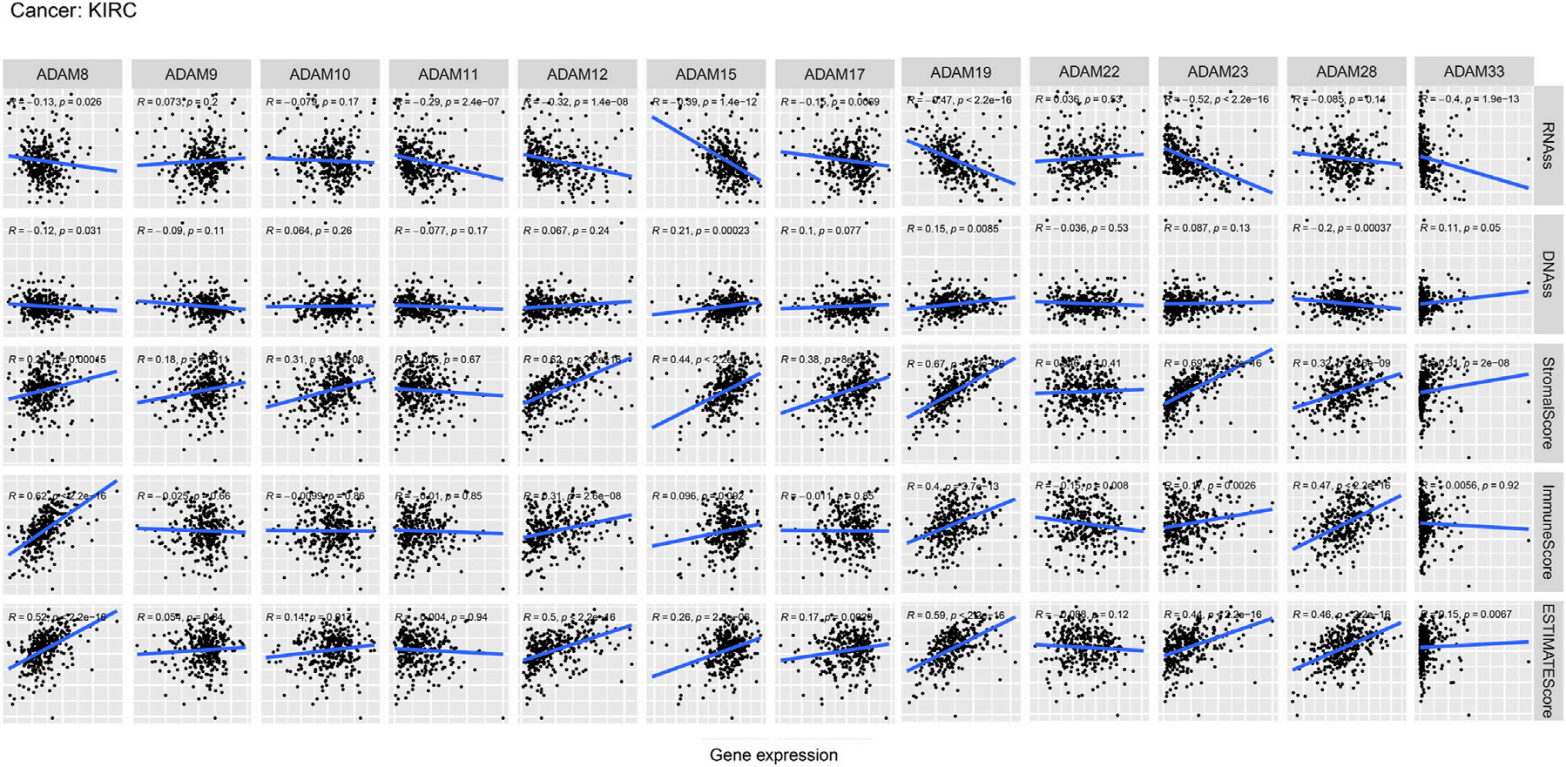
Correlation analysis of ADAM family gene expression with RNAss, DNAss, stromal score, immune score, and ESTIMATE score in KIRC.

We also revealed the relevance between ADAMs and the stromal score, immune score, stemness score, and ESTIMATE score in certain types of cancer (BLCA and KIRC) (Figures 6, 7; Supplementary Table S6) and found that ADAMs had an extensive correlation with the BLCA and KIRC TME as well as DNAss and RNAss.

### 3.4 Association of ADAMs with immune subtypes in pan-cancers

**FIGURE 8:**
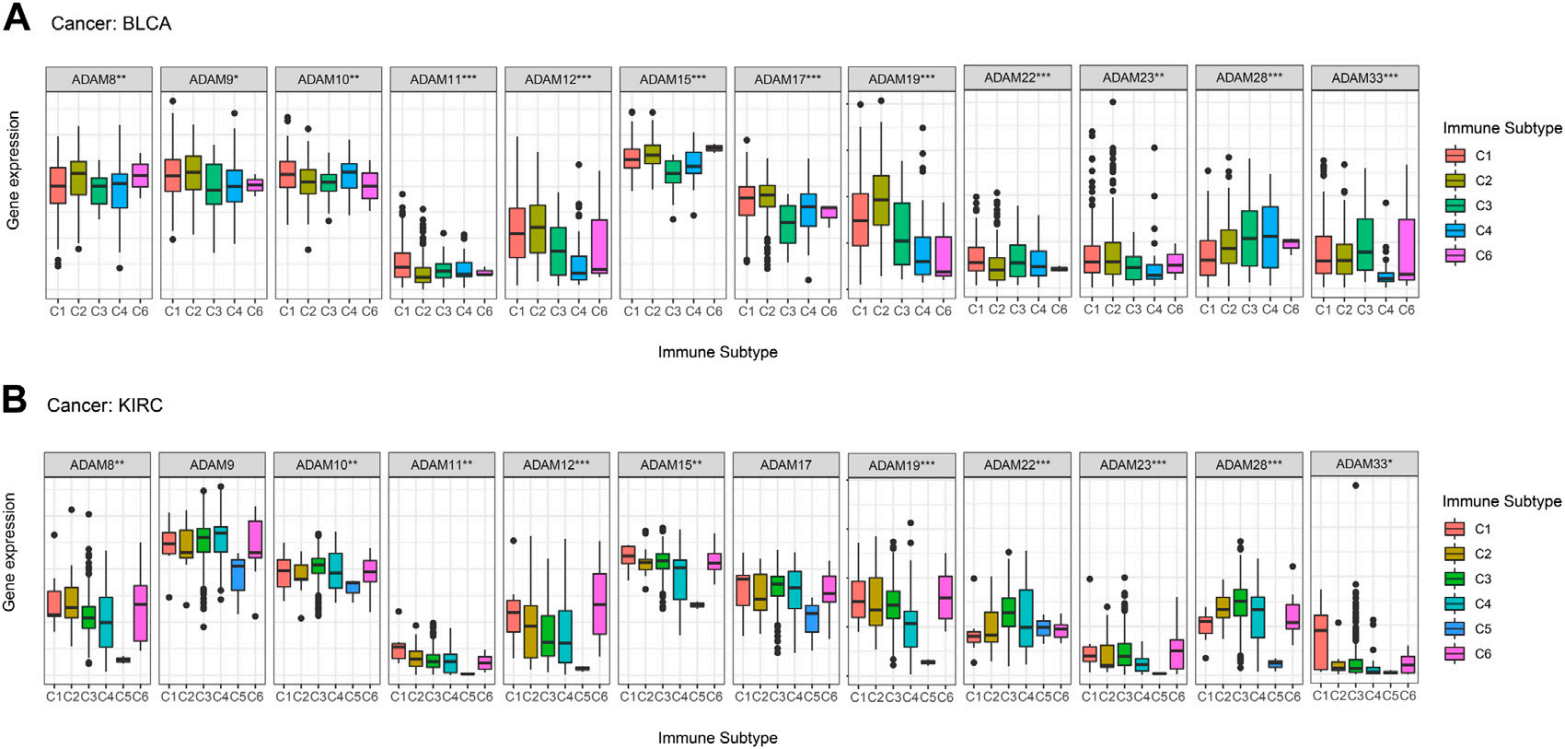
Correlation between ADAM family expression and immune subtypes in BLCA and KIRC. **(A)** ADAM family expression in different immune subtypes in BLCA. **(B)** ADAM family expression in different immune subtypes in KIRC. The X-axis represents the immune subtype, and the Y-axis represents gene expression. C1, wound healing; C2, IFN-γ-dominant; C3, inflammatory; C4, lymphocyte-depleted; C5, immunologically quiet; C6, TGF-β-dominant. *p* < 0.05; **p < 0.01; ***p < 0.001.

Thorsson et al. (2019) put forward six immune subtypes (C1–C6) among 33 pan-cancer types. They performed an extensive immunogenomic analysis of over 10,000 tumours, comprising 33 diverse cancer types and utilising data compiled by TCGA. They identified six immune subtypes: IFN-γ-dominant, wound healing, lymphocyte-depleted, inflammatory, TGF-β-dominant, and immunologically quiet, which showed the different characteristics of immune cell infiltration and prognosis. The immune subtypes were significantly associated with tumour prognosis and genetic and immunomodulatory changes. Thus, we further explored the correlation of ADAMs with the immune subtypes and observed that ADAM8, ADAM10, ADAM11, ADAM12, ADAM15, ADAM19, ADAM22, ADAM23, ADAM28, and ADAM33 were differentially expressed in both BRCA and COAD (Figure 8). Additionally, ADAM19 showed a significantly higher expression in C1–C3 of BLCA and KIRC, whereas ADAM8 was highly expressed in C6 of BLCA and KIRC. Moreover, the expression of the ADAM family in C5 was generally lower than that in other immune subtypes in KIRC. Thus, ADAMs were correlated with these immune subtypes (Figure 8), which improved the prognostic value and potential clinical use of ADAMs.

### 3.5 Association of ADAMs with the outcome of chemotherapy and immunotherapy treatments in the pan-cancer cohort

**FIGURE 9:**
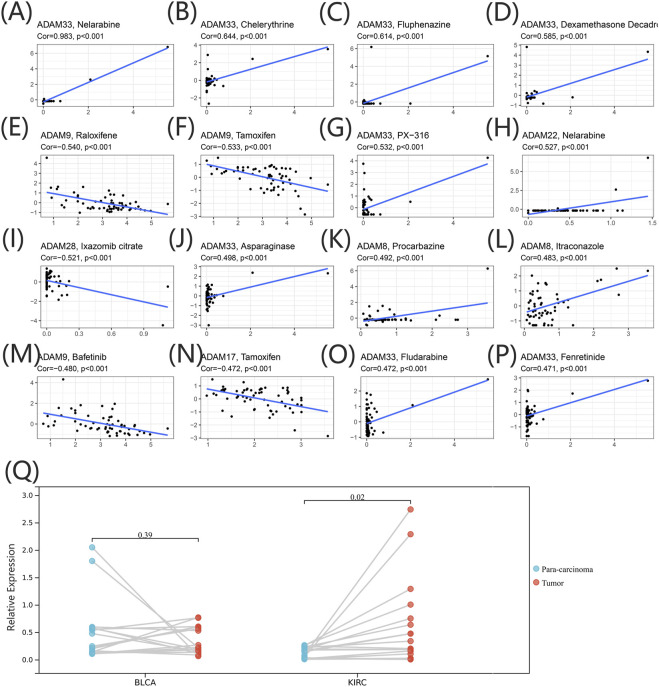
**(A–P)** Drug sensitivity analysis of the ADAM family gene. The X-axis represents the relative sensitivity with certain drugs, and the Y-axis represents the relative expression of ADAMs. **(Q)** Differential expression of ADAM9 in BLCA and KIRC tissues.

We extracted drug sensitivity data from the CellMiner database to determine the correlation between ADAMs and drug sensitivity. The top 16 drugs showing sensitivity to ADAMs are shown in Figure 9 and Supplementary Table S7. Notably, ADAM33 was positively correlated with the sensitivity of nelarabine, chelerythrine, fluphenazine, dexamethasone (Decadron), PX-316, asparaginase, fludarabine, and fenretinide (Figures 9A–D, G, J, O, P). Additionally, raloxifene, tamoxifen, and bafetinib were negatively correlated with ADAM9 (Figures 9E, F, M). Furthermore, nelarabine was positively correlated with ADAM22 (Figure 9H); procarbazine and itraconazole shared a positive correlation with ADAM8 (Figures 9K, L); ADAM28 was negatively correlated with ixazomib citrate (Figure 9I); and ADAM17 was negatively correlated with tamoxifen (Figure 9N). ADAM9 was significantly associated with 82 different drug sensitivities, including tamoxifen, cyclophosphamide, oxaliplatin, and bafetinib. Furthermore, ADAM9 showed significantly different expression between tumour and para-carcinoma tissues in KIRC using qRT-PCR analysis (Figure 9Q). The protein expression of ADAM9 in KIRC using immunohistochemistry revealed results consistent with that of qRT-PCR, wherein a significantly higher expression of ADAM9 in tumour tissues was confirmed. However, the differential expression of ADAM9 in BLCA samples was also observed (Supplementary Figure S2).

**FIGURE 10:**
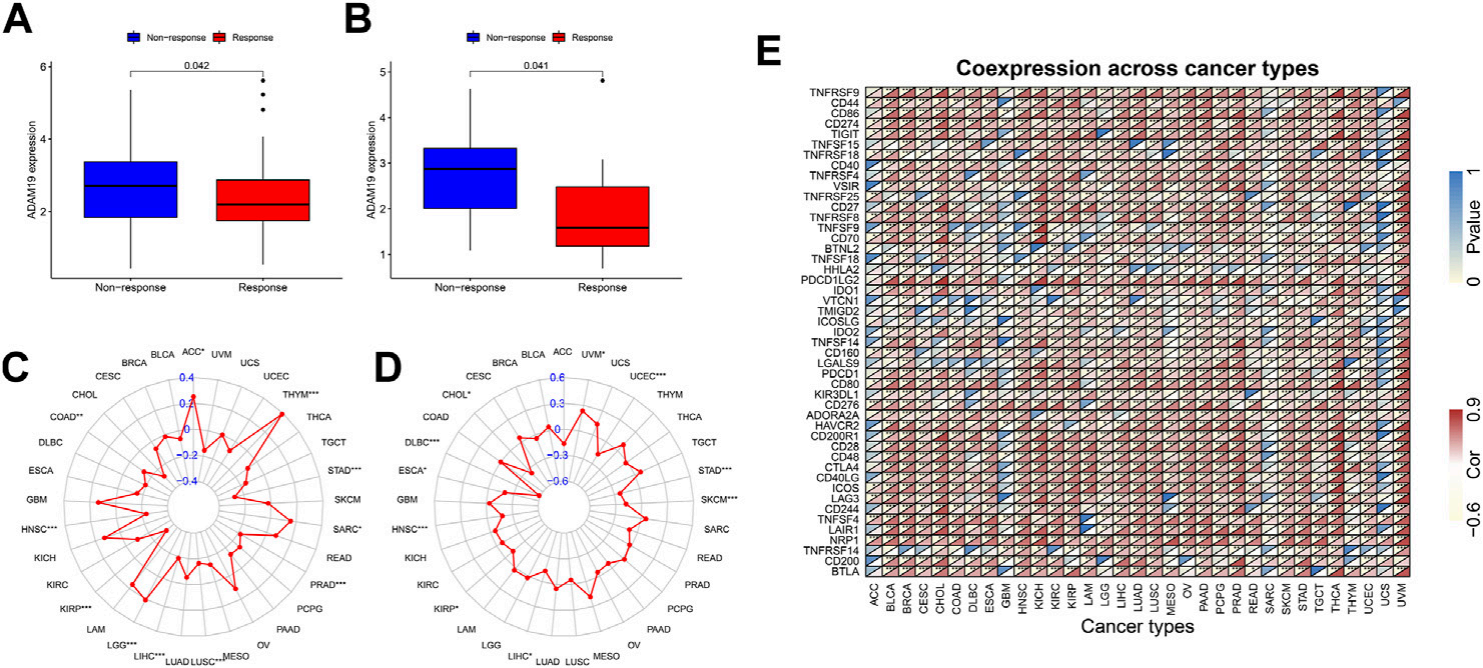
Correlation of immunotherapy outcome, TMB, MSI, and immune activation-related genes with the expression of ADAM19. **(A)** Expression levels of ADAM19 in GSE78220. **(B)** Expression levels of ADAM19 in IMvigor210 **(C)** The relationship between TMB and ADAM19 expression **(D)** The relationship between MSI and ADAM19 expression **(E)** Co-expression of immune activation genes with ADAM19.

We further extracted the expression levels of ADAM19 from the GSE78220 (Figure 10A) and IMvigor210 (Figure 10B) datasets and compared the expression with immune response. ADAM19 was significantly negatively correlated with immunotherapy response.

TMB has been recently considered a potential predictive biomarker of immunotherapy (Picard et al., 2020; Jardim et al., 2021; Rizzo et al., 2021). Meanwhile, MSI has also been reported to be correlated with immunotherapy outcomes (Rizzo et al., 2021). The relationship between TMB and ADAM19 expression was explored (Figure 10C; Supplementary Table S8), and the significant correlation between ADAM19 expression and MSI was detected in various types of cancers, including CHOL, DLBC, ESCA, HNSC, KIRP, LICH, SKCM, STAD, UCEC, and UVM (Figure 10D).

Furthermore, we found the co-expression of immune activation genes and ADAM19 (Figure 10E), revealing a significant correlation of ADAM19 with immune activation genes among almost all 33 types of cancer.

## 4 Discussion

Cancer is the leading cause of death globally (Shams-White et al., 2021; Soerjomataram and Bray, 2021). In 2022, approximately 4.82 million new cancer cases and 3.21 million cancer-related deaths have been reported in China (Xia et al., 2022). Since 2000, the number of cancer-related morbidity and mortality along with their incidence has been increasing annually in China (Wei et al., 2020). Accumulating evidence indicated that proteolytic enzymes, such as MMPs and closely related ADAMs and ADAMTSs, play key roles in cancer initiation and progression (Rocks et al., 2008). Wei et al. (2019) reported that the over-expression of ADAM28 in pancreatic cancer was closely correlated with the regulation of gemcitabine resistance. Similarly, Wang et al. (2011) reported that ADAM10 was significantly associated with lymph node and distant metastasis in gastric cancer. In this study, we focused on the pan-cancer analysis of all ADAMs and explored the differential expression of ADAMs in various types of cancer, as shown in Table 1; we found that both ADAM8 and ADAM12 were located at 10q26. In our results, we also found a significant positive correlation between ADAM8 and ADAM12. Similar results were also observed between ADAM21 and ADAM22. Shimura et al. (2015) revealed that urinary MMP-9/NGAL and urinary ADAM12 are potential non-invasive biomarkers for gastric cancer, including early-stage diseases. Xiao et al. (2012) reported that ADAM17 was an important contributor to prostate cancer invasion according to the shedding of the EGFR ligand TGF-α, which subsequently activates the EGFR–MEK–ERK signalling pathway and induces the over-expression of MMP-2 and MMP-9. Furthermore, Karan et al. (2003) indicated that an inverse expression pattern of ADAM17/TACE and TIMP3 and the regulation of ADAMs with DHT could play an important role in the pathogenesis of prostate cancer. Currently, the most specific therapeutic monoclonal antibody against ADAM17 is D1 (A12), which binds to both the catalytic and disintegrin/cysteine-rich domains of ADAM17 (Tape et al., 2011). It has been proven to be effective in ovarian cancer cells (Richards et al., 2012), breast cancer (Caiazza et al., 2015), and head and neck cancer (Huang et al., 2014). Additionally, ADAM inhibitors can also effectively assist the therapeutic effect of existing monoclonal antibodies. ADAM10 inhibitors have been reported to be protective against HER2-positive breast cancers, which are resistant to Herceptin (trastuzumab) (Rimawi et al., 2015).

The role of ADAMs in tumours remains limited, despite the increasing knowledge of the overall role of ADAMs. In our study, significant differential expression of ADAM8, ADAM9, ADAM10, ADAM11, ADAM12, ADAM15, ADAM17, ADAM19, ADAM22, ADAM23, and ADAM33 was observed among almost all types of cancer. ADAM8 was significantly highly expressed in tumour tissue compared with para-cancerous tissue in all types of cancer, except for KIRC, PRAD, and THCA, indicating ADAM8 could be a significant biomarker for tumours. Notably, the correlation of ADAM8 with tumour progression, metastasis, and chemoresistance in various invasive cancers, including pancreatic cancer (Yu et al., 2019), breast cancer (Conrad et al., 2018), and lung cancer (Ishikawa et al., 2004), has been previously reported. Furthermore, ADAM12 was also generally highly expressed in almost all types of tumour tissues. Wang et al. (2021) reported that ADAM12 inhibition, which was induced by a hypoxia-inducible factor, could effectively downregulate migration and invasion in breast cancer cell lines and also in immuno-deficient mice.

Recently, researchers have been increasingly focusing on the surroundings of solid cancers instead of the tumour itself (Quail and Joyce, 2013; Wu and Dai, 2017; Hinshaw and Shevde, 2019). The surroundings of the cancers are collectively known as the TME which includes immune and stromal cells, and these factors synergistically formed an inflammatory, tumour-promoting, and immuno-suppressive environment which helps cancer cells escape from immune surveillance (Wu and Dai, 2017). In this study, the ESTIMATE algorithm was used to calculate the immune score and stromal score as well as explore the correlation between these scores and ADAM expression. As shown in Figures 5A, B, ADAM8, 19, and 28 showed a significantly positive correlation with both the immune score and stromal score in pan-cancers, indicating that these genes may play an important role in TME development and could be potential immunotherapy targets. Furthermore, we assessed tumour stemness among different ADAMs in cancers using RNAss and DNAss. These results highlighted the negative effect of ADAMs on characterising cancer cells. Furthermore, as shown in Figures 6, 7, a significant positive correlation was observed between ADAM expression, including ADAM8, 9, 12, 19, 23, 28, and 33, whereas ADAM10, 11, and 22 were negatively correlated with the stromal score and immune score.

Although most studies related to ADAM9 focused more on its effect on tumour proliferation, migration, and invasion, various studies have pointed out the value of ADAM9 for tumour drug therapy. In the current study, ADAM9 was significantly associated with 82 different drug sensitivities, including tamoxifen, cyclophosphamide, oxaliplatin, and bafetinib. Liu et al. (2021) revealed that the cisplatin treatment of non-small-cell lung cancer could be promoted by ADAM9 while being negatively regulated by miR-126-5p. Slapak et al. (2021) reported a novel ADAM9-responsive, protease-dependent, drug delivery system for patients with pancreatic ductal adenocarcinoma that can reduce the cytotoxicity of systemic chemotherapy. As for the different results of ADAM9 of PCR and immunohistochemistry in BLCA, it happens occasionally. The transcription and translation of eukaryotic genes were differentially expressed; hence, it is also necessary to determine how the protein level changes.

In terms of cancer treatment, monoclonal antibodies also have their niche population. Monoclonal antibodies have a more specific mode of action and thus cause fewer cytotoxic side effects than low-molecular-weight antibodies. In addition, some monoclonal antibodies can exert anti-cancer activity by inducing antibody-dependent cytotoxicity (Mullooly et al., 2016). Furthermore, in the current study, ADAM19 was negatively correlated with immunotherapy outcomes and strongly correlated with chemotherapy drugs, thus providing a solid theoretical foundation for further research.

## 5 Conclusion

The expression profile of ADAMs in pan-cancers has been demonstrated to correlate with prognosis, tumour microenvironment, treatment outcome, and stemness score. Furthermore, the expression levels of ADAMs in tumour cells are also related to the efficacy of different chemotherapy-related drugs and their response to immunotherapy. These results thus provide a reference for future research on ADAM family genes as potential pan-cancer targets.

## Data Availability

The datasets presented in this study can be found in online repositories. The names of the repository/repositories and accession number(s) can be found in the article/Supplementary Material.
