# Supplementary material for: Pan-cancer analysis of ADAMs: A promising biomarker for prognosis and response to chemotherapy and immunotherapy
Source: Front Genet. 2023 Apr 4;14:1105900. doi: 10.3389/fgene.2023.1105900 (PMC10110990; doi:10.3389/fgene.2023.1105900)
Supplement: Supplementary file 1 [file Presentation1.zip › Supplementary File Captions.DOCX]

Figure S1. ADAM family expression levels in different cancer and para-carcinoma tissue

Figure S2. Immunohistochemical results of ADAM9 in BLCA and KIRC

Table S1: 20 different ADAM genes

Table S2: expression matrix of ADAM genes

Table S3. The immunotherapy outcome of the samples in the GES78220 dataset and IMvigor210 dataset.

Table S4. P value of the KM survival curves of ADAM family in pan-cancer

Table S5. Coefficient and the p value of the correlation between ADAM family and stromal score/immune score/ESTIMATE score/RNAss/DNAss.

Table S6. Coefficient and the p value of the correlation between ADAM family and stromal score/immune score/ESTIMATE score/RNAss/DNAss in BLCA and KIRC.

Table S7. Coefficient and the p value of the drug sensitivity analysis of ADAM family gene.

Table S8. Coefficient and the p value of the correlation between ADAM family and TMB/MSI in pan-cancer.
